# Ultrasound-assisted modified paramedian approach vs. traditional paramedian approach for spinal anesthesia in lower limb surgery: a single-center randomized controlled trial

**DOI:** 10.3389/fmed.2026.1903864

**Published:** 2026-09-03

**Authors:** Wudong Zhuang, Xiaoyuan Li, Jianhua Wang, Ruizhao Lyu, Shan Wang, Rui Liu, Yang Bai

**Affiliations:** 1 Department of Anesthesiology and Perioperative Medicine, Hebei Cangzhou Hospital of Integrated Traditional Chinese Medicine and Western Medicine, Cangzhou, Hebei Province, China; 2 Department of Anesthesiology and Perioperative Medicine, Hebei Province Key Laboratory of Integrated Traditional and Western Medicine in Neurological Rehabilitation, Cangzhou, Hebei Province, China; 3 Department of Anesthesiology and Perioperative Medicine, Hebei Key Laboratory of Integrated Traditional and Western Medicine in Osteoarthrosis Research (Preparing), Cangzhou, Hebei Province, China; 4 Department of Anesthesiology, Cangzhou People’s Hospital, Cangzhou, Hebei Province, China

**Keywords:** modified paramedian approach, needle placement, randomized controlled trial, spinal anesthesia, Ultrasound-assisted

## Abstract

**Study objective:**

This randomized controlled trial aimed to determine whether an ultrasound-assisted modified paramedian approach could improve procedural outcomes compared with an ultrasound-assisted traditional paramedian approach for spinal anesthesia in patients undergoing lower limb surgery.

**Design:**

Prospective, randomized, controlled clinical trial.

**Setting:**

Operating rooms of Hebei Cangzhou Hospital of Integrated Traditional Chinese Medicine and Western Medicine, China.

**Patients:**

A total of 116 patients aged 18–90 years, with a body mass index of 18–30 kg/m^2^ and ASA physical status I–III, scheduled for lower limb surgery requiring spinal anesthesia were enrolled. Patients with contraindications to spinal anesthesia, previous lumbar surgery, scoliosis, or pregnancy were excluded.

**Interventions:**

Patients were randomly assigned in a 1:1 ratio using a computer-generated random allocation sequence with concealed allocation to receive either ultrasound-assisted traditional paramedian spinal anesthesia (Group C, *n* = 58) or ultrasound-assisted modified paramedian spinal anesthesia (Group M, *n* = 58).

**Main results:**

Compared with Group C, Group M required fewer needle passes to achieve successful cerebrospinal fluid (CSF) flow [median [IQR]: 1 [1–2] vs. 2 [1–3], *P* = 0.006] and demonstrated a higher first-pass success rate [37/58 [63.79%] vs. 25/58 [43.10%], *P* = 0.026]. Group M also showed a shorter duration of the spinal procedure [12.5 [9.0–20.0] s vs. 15.0 [10.0–32.25] s, *P* = 0.028] and shorter total procedure time [76.5 [65.0–89.25] s vs. 82.0 [72.0–112.75] s, *P* = 0.031]. The distribution of patient satisfaction scores differed between groups, with a higher proportion of patients reporting being “very satisfied” in Group M (87.93% vs. 70.69%, *P* = 0.040). The incidence of procedure-related and postoperative adverse events was comparable between groups, and no serious complications occurred.

**Conclusions:**

The ultrasound-assisted modified paramedian approach was associated with fewer needle passes, improved first-pass success, and higher patient satisfaction ratings compared with the traditional paramedian approach, while without increasing short-term procedure-related adverse events. Further multicenter studies are warranted to determine whether these findings can be generalized to broader patient populations.

**Clinical Trial Registration:** https://www.chictr.org.cn/showproj.html?proj=203874, identifier ChiCTR2300075567.

## Introduction

Spinal anesthesia is widely used for lower limb surgery because it provides effective analgesia, reliable sensory and motor blockade, and favorable perioperative outcomes. However, successful spinal anesthesia depends on accurate identification of the target intervertebral space and precise advancement of the spinal needle. Technical challenges remain common, particularly in patients with difficult spinal anatomy or when the procedure is performed by clinicians with limited experience. Multiple needle attempts or repeated needle redirection may prolong procedural time, increase patient discomfort, and adversely affect patient satisfaction. Ultrasound guidance has increasingly been incorporated into neuraxial anesthesia practice as an adjunct technique. By improving visualization of spinal anatomical structures and facilitating identification of the target interlaminar space, ultrasound-assisted approaches may enhance needle placement accuracy and procedural success (1–4).

The traditional paramedian approach is frequently used for spinal anesthesia, particularly when the midline approach is difficult or unsuitable because of anatomical variations (5–11). However, successful paramedian puncture requires accurate estimation of the needle trajectory and sufficient technical experience. Inappropriate needle direction or repeated adjustments may increase the number of needle passes, prolong procedure duration, and contribute to patient discomfort (12). Ultrasound-assisted localization allows more precise identification of anatomical landmarks and the intended puncture site, which may facilitate more standardized needle placement (13).

Several studies have explored modified paramedian techniques to further optimize needle placement. Zeng et al. reported that an ultrasound-assisted modified paramedian technique improved puncture-related outcomes in elderly patients undergoing spinal anesthesia, whereas Qu et al. evaluated a modified ultrasound-assisted paramedian technique in elderly patients undergoing combined spinal-epidural anesthesia (14, 15). However, previous studies mainly involved elderly populations or specific neuraxial anesthesia procedures. Whether modification of the puncture trajectory after ultrasound-assisted localization provides additional advantages over the ultrasound-assisted traditional paramedian approach in patients undergoing spinal anesthesia for lower limb surgery remains unclear. Further investigation is therefore required to determine whether optimization of the needle insertion pathway can improve procedural performance.

This randomized controlled trial was designed to compare the ultrasound-assisted modified paramedian approach with the ultrasound-assisted traditional paramedian approach in patients undergoing lower limb surgery. We hypothesized that the ultrasound-assisted modified paramedian approach would reduce the number of needle passes required to achieve successful cerebrospinal fluid (CSF) flow while maintaining comparable safety outcomes. The primary outcome was the number of needle passes required to obtain free CSF flow, which was selected as an indicator of technical difficulty during spinal needle advancement. Secondary outcomes were assessed to evaluate procedural reliability, efficiency, patient experience, and safety, including first-pass success rate, number of skin punctures, ultrasound-assisted localization time, duration of the spinal procedure, total procedure time, patient satisfaction, ultrasound image quality, and procedure-related adverse events.

## Methods

### Study design and participants

This prospective, randomized controlled trial was approved by the Institutional Review Board of Hebei Cangzhou Hospital of Integrated Traditional Chinese Medicine and Western Medicine (approval number: CZX2023-KY-080.1; August 28, 2023). Written informed consent was obtained from all participants before enrollment. The study was prospectively registered in the Chinese Clinical Trial Registry (ChiCTR2300075567) on September 8, 2023, before patient enrollment, and was conducted in accordance with the Consolidated Standards of Reporting Trials (CONSORT) guidelines.

Eligible participants were patients aged 18–90 years who were scheduled to undergo lower limb surgery requiring subarachnoid anesthesia. Patients of both sexes were eligible, and those with American Society of Anesthesiologists (ASA) physical status I–III were included according to the predefined criteria. Although patients with ASA physical status I–III were eligible for enrollment, all included participants were ultimately classified as ASA II because of the clinical characteristics of the recruited surgical population.

Patients were excluded if they had contraindications to subarachnoid anesthesia, severe cardiovascular or pulmonary diseases, a history of lumbar spine surgery, spinal scoliosis, pregnancy, or lactation.

### Randomization

Patients were randomly assigned in a 1:1 ratio to receive either the ultrasound-assisted traditional paramedian approach (Group C) or the ultrasound-assisted modified paramedian approach (Group M). Randomization was performed using a computer-generated allocation sequence with a block size of 4. The sequence was generated by an independent statistician who was not involved in patient enrollment, intervention administration, or outcome assessment.

Allocation concealment was maintained using sequentially numbered, opaque, sealed envelopes. The envelopes were opened by the anesthesiologist after patient positioning and immediately before the procedure.

Patients were blinded to group allocation during the procedure and postoperative assessment. Because of the nature of the intervention, blinding of the anesthesiologist performing the procedure was not feasible. However, all procedures were performed by the same experienced anesthesiologist, who had completed at least 50 ultrasound-assisted paramedian spinal anesthesia procedures and passed a standardized competency assessment, thereby minimizing variability related to differences in operator technique.

All procedures were video-recorded. Two independent investigators who were not involved in the procedures and were blinded to group allocation reviewed the recordings to assess the number of needle passes and procedure duration. Patient satisfaction was evaluated using standardized questions administered by investigators who were not involved in the anesthesia procedure and were unaware of group allocation. Postoperative follow-up and adverse event assessment were performed by independent investigators blinded to treatment assignment.

Outcome data were recorded using numerical codes rather than group identifiers. Statistical analyses were performed using coded datasets, and group allocation was revealed only after completion of the analysis.

### Procedures

Before study initiation, the anesthesiologist responsible for all procedures had completed more than 50 supervised ultrasound-assisted paramedian spinal anesthesia procedures and passed a standardized competency assessment. The anesthesiologist had 5 years of clinical anesthesia experience and had performed more than 500 spinal anesthesia procedures before the initiation of the study. Since operator blinding was not feasible due to the intervention characteristics, all procedures were performed by the same anesthesiologist to minimize inter-operator variability.

Upon arrival in the operating room, standard monitoring, including non-invasive blood pressure measurement, three-lead electrocardiography, and pulse oximetry, was initiated. Supplemental oxygen was administered through a face mask at a flow rate of 1–2 L/min, and peripheral intravenous access was established. Patients were then positioned in the lateral decubitus position with the surgical side facing upward under the guidance of the anesthesiologist.

Before ultrasound-assisted localization, lumbar surface landmarks were palpated in both groups. The difficulty of landmark palpation was assessed using a previously reported 4-point scale (6).

### Ultrasound-assisted modified paramedian technique

In Group M, a standardized three-step ultrasound-assisted localization protocol was applied using a 2–8 MHz curved array transducer (AENUE R2; GE Medical Systems Ultrasound & Primary Care Diagnostics LLC, WI, USA).

#### Identification of the target interlaminar space

**Figure 1:**
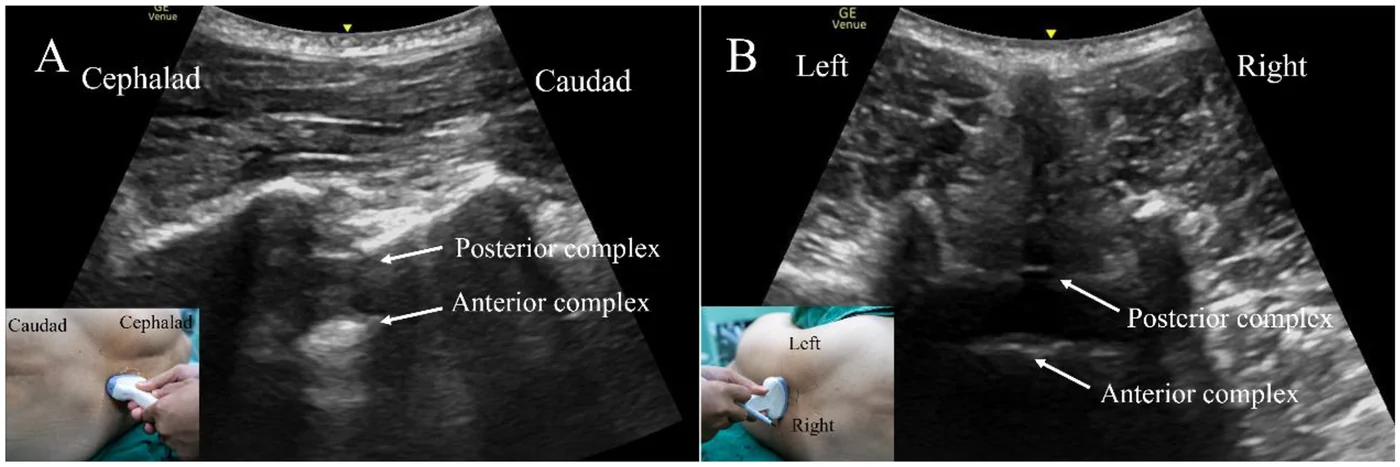
Ultrasonographic views used for preprocedural spinal localization. **(A)** Parasagittal oblique (PSO) view of the lumbar spine. The inset in the lower left corner illustrates the corresponding ultrasound probe position for obtaining the PSO view. **(B)** Transverse median (TM) view of the lumbar spine. The inset in the lower left corner illustrates the corresponding ultrasound probe position for identifying the ultrasound-defined spinal midline.

The ultrasound probe was positioned in the parasagittal oblique (PSO) plane (Figure 1A), approximately 1–2 cm lateral to the midline. Scanning was initiated from the sacrum and progressed cranially using a sequential counting method to identify the interlaminar spaces from L5–S1 to L2–L3. The L3/4 or L2/3 interlaminar space was selected according to ultrasound image quality and visualization of the anterior and posterior complexes (Figure 1A).

The upper edge of the inferior lamina was positioned at the center of the ultrasound screen. Under M-mode assistance, a skin marking line perpendicular to the midpoint of the probe's long axis was drawn to indicate the skin projection of the target interlaminar space.

#### Identification of the spinal midline

The probe was positioned in the transverse median (TM) plane to evaluate spinal anatomical structures (Figure 1B). The probe angle was adjusted to obtain the optimal ultrasound image. The midpoint of the long edge of the probe was marked on the skin surface to indicate the ultrasound-defined spinal midline.

#### Determination of the modified puncture point

**Figure 2:**
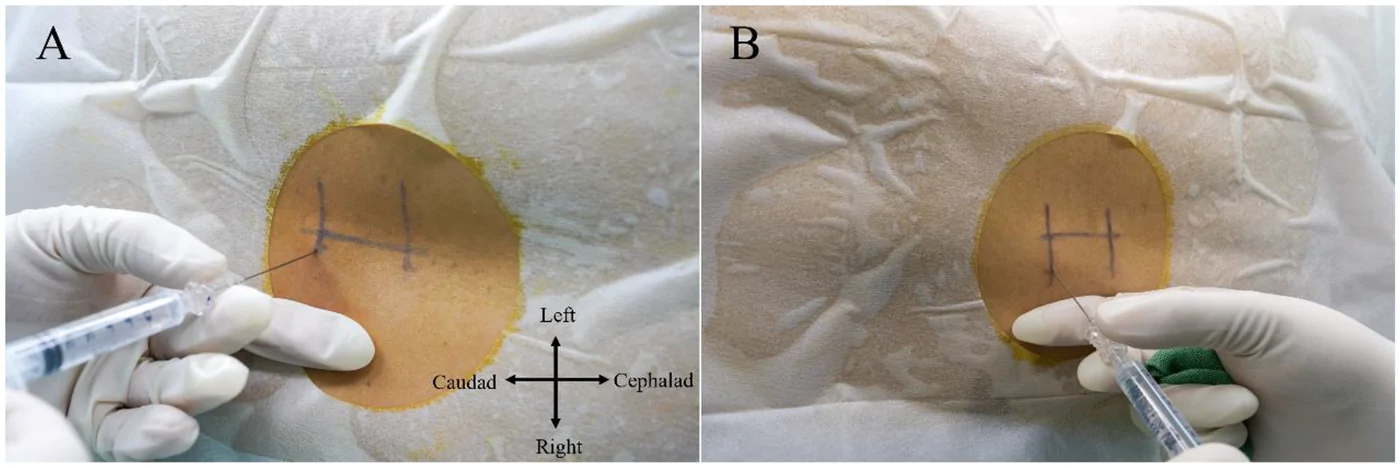
Comparison of needle insertion techniques between the modified and traditional paramedian approaches. **(A)** Ultrasound-assisted modified paramedian approach. The puncture point was located 0.5 cm lateral to the ultrasound-defined spinal midline on the non-operative side, and the spinal needle was advanced with a near-perpendicular trajectory relative to the skin surface. **(B)** Ultrasound-assisted traditional paramedian approach. The puncture point was located 1.5 cm lateral to the ultrasound-defined spinal midline on the non-operative side, and the spinal needle was advanced using the conventional oblique paramedian trajectory. Red dotted lines indicate the intended needle trajectories according to the predefined techniques.

The intersection of the two skin marking lines was defined as Point “O”, representing the skin projection of the target interlaminar space and spinal midline. In Group M, the puncture point was located 0.5 cm lateral to Point “O” on the non-operative side of the midline. The spinal needle was then advanced according to the modified paramedian technique (Figure 2A).

### Ultrasound-assisted traditional paramedian technique

In Group C, the same three-step ultrasound-assisted localization protocol was applied using the same ultrasound transducer.

#### Identification of the target interlaminar space

The probe was positioned in the PSO plane (Figure 1A), approximately 1–2 cm lateral to the midline. Scanning was initiated from the sacrum and progressed cranially using sequential counting to identify the interlaminar spaces from L5–S1 to L2–L3. The L3/4 or L2/3 interlaminar space was selected according to ultrasound image quality and visualization of the anterior and posterior complexes (Figure 1A).

The midpoint of the target interlaminar space was positioned at the center of the ultrasound screen, and a skin marking line perpendicular to the midpoint of the probe's long axis was drawn under M-mode assistance to indicate the skin projection of the target interlaminar space.

#### Identification of the spinal midline

The probe was positioned in the TM plane to evaluate spinal anatomical structures (Figure 1B). The probe angle was adjusted to obtain the optimal image. The midpoint of the long edge of the probe was marked to indicate the ultrasound-defined spinal midline.

#### Determination of the traditional paramedian puncture point

The intersection of the two marking lines was defined as Point “O”. In Group C, the puncture point was located 1.5 cm lateral to Point “O” on the non-operative side of the midline. The spinal needle was inserted using the conventional paramedian trajectory at an approximately 75° angle (Figure 2B).

### Spinal anesthesia procedure

Strict aseptic techniques were applied throughout the procedure in both groups. Spinal anesthesia was performed using a needle-through-needle technique with a 0.7 × 90 mm disposable anesthesia puncture kit (Henan Haisen Medical Equipment Co., Ltd., Henan, China). After confirmation of free cerebrospinal fluid (CSF) reflux, 0.5% bupivacaine (8–15 mg) was administered intrathecally.

If successful CSF access was not achieved after three needle passes, a different interlaminar space was selected for further attempts. Failure to obtain CSF access after attempts at two different interlaminar spaces was defined as technical failure, and rescue procedures were performed according to the clinical judgment of the attending anesthesiologist.

All procedures were completed by the same anesthesiologist. Sensory block level was assessed by loss of cold sensation 15 min after intrathecal injection. A sensory block level below T10 at this time point was considered spinal anesthesia failure, and subsequent management was determined by the attending anesthesiologist. All technical failures and spinal anesthesia failures, if present, were recorded as study outcomes.

Ultrasound image quality and patient satisfaction related to the procedure were assessed according to predefined criteria. All procedures were video-recorded. Procedural parameters, including the number of needle passes and procedure duration, were recorded by a research assistant and independently verified by two investigators based on the video recordings. Postoperative follow-up was performed within 48 h after surgery to evaluate potential complications and adverse events.

### Study outcomes

#### Primary outcome

The primary outcome was the number of needle passes required to obtain free-flowing cerebrospinal fluid (CSF). A needle pass was defined as each withdrawal or redirection of the needle after the initial insertion until successful CSF flow was achieved. The number of needle passes was independently assessed by two investigators blinded to group allocation based on recorded videos.

#### Secondary outcomes

Secondary outcomes included first-pass success rate, number of skin punctures, ultrasound-assisted localization time, duration of the spinal procedure, total procedure time, patient satisfaction related to the procedure, Difficulty of Palpation, and ultrasound image quality.

First-pass success was defined as successful CSF acquisition following a single needle insertion without needle withdrawal or redirection.

The number of skin punctures was defined as the number of separate skin entry points required to achieve free CSF flow, regardless of subsequent needle manipulation or redirection.

Procedure time consisted of ultrasound-assisted localization time, duration of the spinal procedure, and total procedure time. Ultrasound-assisted localization time was defined as the interval from initial contact of the ultrasound probe with the patient's skin to completion of marking the needle insertion point. The duration of the spinal procedure was defined as the interval from needle contact with the skin to confirmation of free CSF flow. Total procedure time was calculated as the sum of ultrasound-assisted localization time and duration of the spinal procedure.

Patient satisfaction related to the procedure was assessed 1 min after intrathecal injection using a 5-point ordinal scale: 1 = very unsatisfied, 2 = unsatisfied, 3 = neutral, 4 = satisfied, and 5 = very satisfied (15). The scale was analyzed as an ordinal variable. Although this scale has been applied in previous procedural studies, it has not been validated as a patient-reported outcome instrument.

Difficulty of Palpation was assessed using a 4-point scale: grade 1, easy, defined as the iliac crest or spinous process being visible to the eye; grade 2, moderate, defined as the iliac crest or spinous process being palpable with light pressure; grade 3, difficult, defined as the iliac crest or spinous process being palpable only with firm pressure; and grade 4, very difficult, defined as the iliac crest or spinous process remaining impalpable even with firm pressure (6).

Ultrasound image quality was evaluated for both the parasagittal oblique (PSO) view and transverse median (TM) view using a 3-point ordinal scale: 1 = clear, with both anterior and posterior complexes visible; 2 = moderate, with either anterior or posterior complex visible; and 3 = poor, with neither anterior nor posterior complex visible.

#### Adverse events

Adverse events were recorded during the procedure and within 48 h after surgery.

Intraoperative adverse events included vascular puncture and paresthesia during needle advancement. Procedure-related failure outcomes included failure to obtain CSF flow and spinal anesthesia failure. Postoperative adverse events included post-dural puncture headache, lower back pain, neurological symptoms, and other clinically relevant complications.

#### Statistical methods

Sample size calculation was performed using PASS 15.0 software. Based on previous literature, the mean number of needle passes required to obtain free CSF flow with the ultrasound-assisted traditional paramedian approach was 1.9 ± 1.8 (9). A reduction of approximately one needle pass was considered clinically meaningful and was used as the expected between-group difference for sample size estimation. Assuming a two-sided significance level of 0.05, statistical power of 80%, and a 10% anticipated dropout rate, 58 patients were required in each group, resulting in a total sample size of 116 participants.

All statistical analyses were performed using SPSS version 25.0 (IBM Corp., Armonk, NY, USA). Continuous variables were assessed for distribution normality using histograms and the Kolmogorov–Smirnov test. Normally distributed continuous variables were expressed as mean ± standard deviation (SD), whereas non-normally distributed variables were presented as median [interquartile range (IQR)]. Categorical variables were summarized as number (percentage).

Non-normally distributed continuous variables, including the number of needle passes, ultrasound-assisted localization time, duration of the spinal procedure, and total procedure time, were compared between groups using the Mann–Whitney *U* test. Categorical variables, including first-pass success rate, number of skin punctures, patient satisfaction, Difficulty of Palpation, ultrasound image quality, and adverse events, were analyzed using the chi-square test or Fisher's exact test, as appropriate. A two-tailed *P* value < 0.05 was considered statistically significant.

## Results

**Figure 3:**
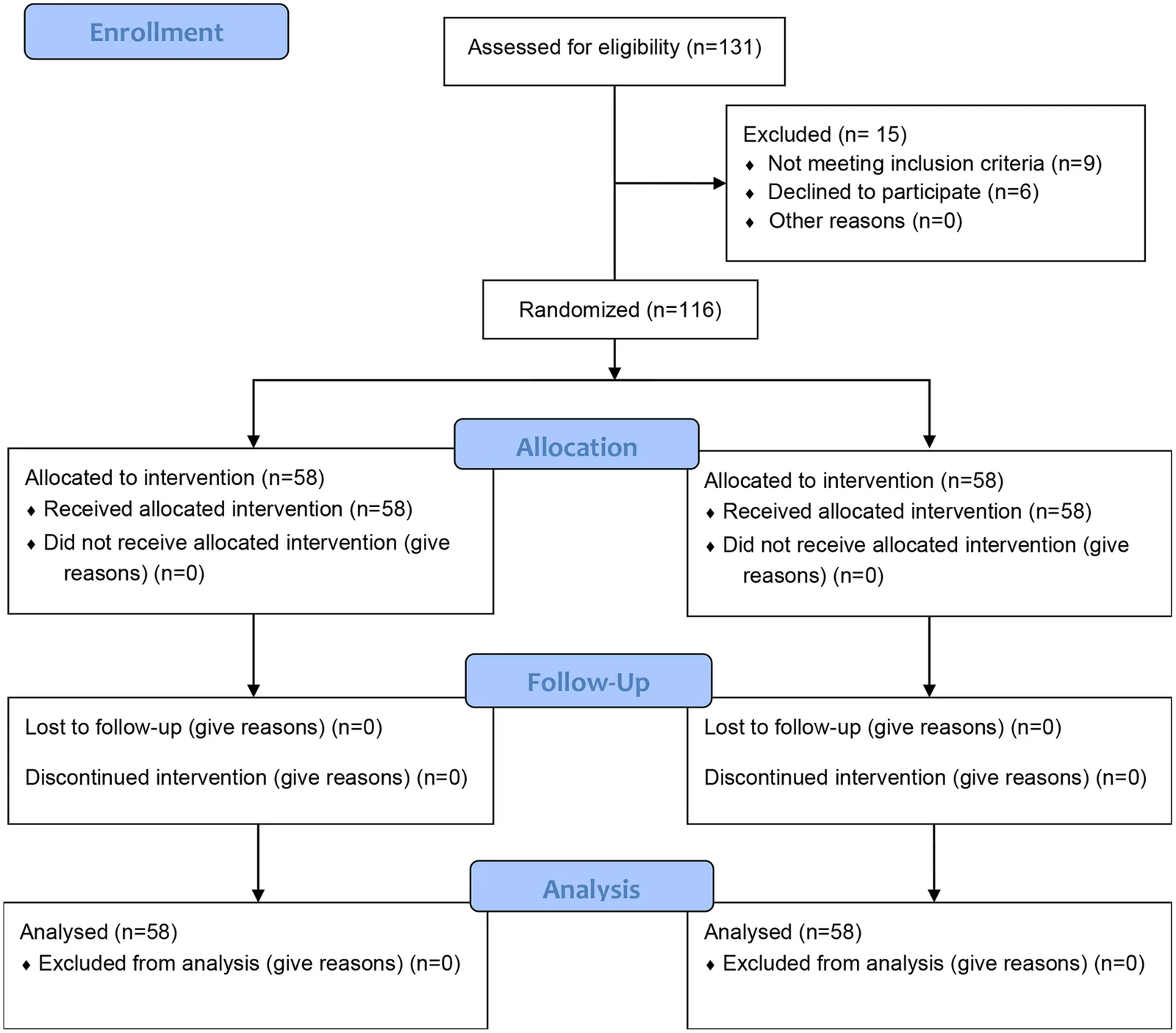
CONSORT flow diagram of patient recruitment and analysis. CONSORT, Consolidated Standards of Reporting Trials.

As shown in the CONSORT flow diagram (Figure 3), 131 patients were assessed for eligibility. Fifteen patients were excluded because they did not meet the predefined eligibility criteria. No patients withdrew from the study or were lost to follow-up. No technical failures or spinal anesthesia failures occurred in either group according to the predefined criteria, and no rescue procedures, including conversion to general anesthesia or alternative regional anesthesia, were required. Finally, 116 patients completed the study and were included in the final analysis, with 58 patients assigned to each group.

Baseline characteristics, including sex, age, height, weight, BMI, and ASA physical status, were comparable between the two groups (Table 1).

**Table 1:** Baseline characteristics of patients in Group C and Group M.

Characteristic	Group C (*n* = 58)	Group M (*n* = 58)	*P* value
Age (years), mean ± SD	46.41 ± 12.80	46.40 ± 12.60	0.994
Sex, *n* (%)			0.126
Male	32 (55.17)	40 (68.97)	
Female	26 (44.83)	18 (31.03)	
Height (cm), median (IQR)	168.00 (159.75–174.25)	170.00 (165.00–175.00)	0.261
Weight (kg), mean ± SD	72.14 ± 12.99	73.13 ± 11.12	0.660
BMI (kg/m^2^), median (IQR)	25.73 (23.40–28.03)	25.50 (22.90–28.86)	0.903
ASA physical status, *n* (%)			1.000
I	0 (0)	0 (0)	
II	58 (100)	58 (100)	
III	0 (0)	0 (0)	

Data are presented as mean ± standard deviation (SD), median [interquartile range (IQR)], or number (percentage), as appropriate. Group C, conventional paramedian approach; Group M, modified paramedian approach.

BMI, body mass index; ASA, American Society of Anesthesiologists physical status classification.

The primary outcome differed significantly between groups. Compared with Group C, Group M required fewer needle passes to achieve free-flowing CSF [median [IQR]: 1 [1–2] vs. 2 [1–3], *P* = 0.006]. The first-pass success rate was higher in Group M than in Group C [37/58 [63.79%] vs. 25/58 [43.10%], *P* = 0.026].

The duration of the spinal procedure was shorter in Group M than in Group C [12.5 [9.0–20.0] s vs. 15.0 [10.0–32.25] s, *P* = 0.028], and the total procedure time was also reduced [76.5 [65.0–89.25] s vs. 82.0 [72.0–112.75] s, *P* = 0.031]. Patient satisfaction scores differed significantly between groups, with a greater proportion of patients in Group M reporting a score of 5 (“very satisfied”) (87.93% vs. 70.69%, *P* = 0.040).

No significant differences were observed between groups in the number of skin punctures, ultrasound-assisted localization time, puncture level, bupivacaine dosage, or sensory block level (Table 2).

**Table 2:** Comparison of procedural outcomes between the two groups.

Variable	Group C (*n* = 58)	Group M (*n* = 58)	Effect estimate (95% CI)	*P* value
Primary outcome				
Number of needle passes, median (IQR)	2 (1–3)	1 (1–2)	Median difference: 1.00 (0.00–1.00)	0.006
Key secondary outcome				
First-pass success, *n* (%)	25 (43.10)	37 (63.79)	RR: 1.48 (1.03–2.12)	0.026
Continuous secondary outcomes				
Ultrasound-assisted localization time (s), median (IQR)	65.00 (5.75–71.50)	61.50 (55.00–71.00)	Median difference: 3.50 (−3.00–6.00)	0.497
Duration of spinal procedure (s), median (IQR)	15.00 (10.00–32.25)	12.50 (9.00–20.00)	Median difference: 2.50 (0.00–7.00)	0.028
Total procedure time (s), median (IQR)	82.00 (72.00–112.75)	76.50 (65.00–89.25)	Median difference: 5.50 (−1.00–16.00)	0.031
Bupivacaine dosage (mg), median (IQR)	15.00 (15.00–15.00)	15.00 (15.00–15.00)	Median difference: 0.00 (0.00–0.00)	0.946
Anesthesia plane, median (IQR)	8.00 (6.00–9.00)	7.50 (6.00–9.00)	Median difference: 0.50 (0.00–1.00)	0.308
Other categorical outcomes				
Number of skin punctures, *n* (%)				0.675
1	54 (93.10)	56 (96.55)		
2	4 (6.90)	2 (3.45)		
Patient satisfaction, *n* (%)				0.040
1 Very unsatisfied	0 (0)	0 (0)		
2 Unsatisfied	0 (0)	1 (1.72)		
3 Neutral	3 (5.17)	0 (0)		
4 Satisfied	14 (24.14)	6 (10.34)		
5 Very satisfied	41 (70.69)	51 (87.93)		
Puncture level, *n* (%)				0.769
L3–4	52 (89.66)	51 (87.93)		
L2–3	6 (10.34)	7 (12.07)		

Data are presented as median [interquartile range (IQR)] for continuous variables and number (percentage) for categorical variables. Effect estimates are presented as relative risk (RR) with 95% confidence intervals (CI) for categorical outcomes and median differences with 95% CI for continuous outcomes. RR values were calculated as Group M relative to Group C. Continuous variables were analyzed using the Mann–Whitney *U* test, and categorical variables were analyzed using the chi-square test or Fisher's exact test, as appropriate. A two-sided *P* value < 0.05 was considered statistically significant.

Group C, conventional paramedian approach; Group M, modified paramedian approach.

The distribution of lateral puncture sides and Difficulty of Palpation grades did not differ significantly between groups. The distribution of ultrasound image quality grades was also comparable between groups for both the TM and PSO views (Table 3).

**Table 3:** Comparison of puncture difficulty assessment and ultrasound image quality between the two groups.

Variable	Group C (*n* = 58)	Group M (*n* = 58)	*P* value
Left/right puncture side, *n* (%)			0.575
Left side	31 (53.4)	34 (58.6)	
Right side	27 (46.6)	24 (41.4)	
Difficulty of palpation, *n* (%)			0.666
1 Easy	15 (25.86)	17 (29.31)	
2 Moderate	30 (51.72)	29 (50.00)	
3 Difficult	12 (20.69)	9 (15.52)	
4 Very difficult	1 (1.72)	3 (5.17)	
Ultrasound image quality			
TM view, *n* (%)			0.800
1 Clear	15 (25.86)	12 (20.69)	
2 Moderate	38 (65.52)	41 (70.69)	
3 Poor	5 (8.62)	5 (8.62)	
PSO view, *n* (%)			0.826
1 Clear	44 (75.86)	45 (77.59)	
2 Moderate	14 (24.14)	13 (22.41)	
3 Poor	0 (0)	0 (0)	

Data are presented as number (percentage). Puncture difficulty was assessed using a 4-point scale: 1 (easy), 2 (moderate), 3 (difficult), and 4 (very difficult). Ultrasound image quality was graded using a 3-point scale: 1 (clear), 2 (moderate), and 3 (poor).

TM, transverse median; PSO, parasagittal oblique.

As illustrated in Figure 1, the PSO and TM views were used complementarily to identify the target interlaminar space and ultrasound-defined spinal midline, respectively. In a supplementary analysis, ultrasound image quality differed significantly between PSO and TM views (Bowker's test of symmetry, *P* < 0.001). Compared with the TM view, the PSO view demonstrated a higher proportion of clear images (Supplementary Table S1).

The incidence of intraoperative adverse events, including puncture paresthesia and blood return in the puncture needle, was comparable between groups. No patients experienced lower limb sensory abnormalities or post-dural puncture headache during follow-up.

Lower back pain was reported in 4 patients in Group C and 2 patients in Group M, with no statistically significant difference between groups (*P* = 0.675). No serious complications occurred within the 48 h follow-up period (Table 4).

**Table 4:** Comparison of intraoperative and postoperative adverse events within 48 H between the two groups.

Variable	Group C (*n* = 58)	Group M (*n* = 58)	*P* value
Puncture paresthesia, *n* (%)	6 (10.34)	2 (3.45)	0.272
Blood in puncture needle, *n* (%)	1 (1.72)	2 (3.45)	1.000
Lower limb sensory abnormality, *n* (%)	0 (0)	0 (0)	—
Post-dural puncture headache, *n* (%)	0 (0)	0 (0)	—
Lower back pain, *n* (%)	4 (6.90)	2 (3.45)	0.675

Data are presented as number (percentage). Puncture paresthesia was defined as transient abnormal sensations experienced during needle insertion. Lower limb sensory abnormality was defined as persistent sensory changes after the procedure. Adverse events were assessed within 48 h after surgery. Comparisons were performed using chi-square test or Fisher's exact test, as appropriate.

## Discussion

In this randomized controlled trial, we compared two ultrasound-assisted paramedian approaches for spinal anesthesia in patients undergoing lower limb surgery. The ultrasound-assisted modified paramedian approach resulted in fewer needle passes and a higher first-pass success rate compared with the ultrasound-assisted traditional paramedian approach, while maintaining comparable short-term safety outcomes. These findings suggest that modification of the puncture pathway may provide additional procedural benefits after ultrasound-assisted anatomical localization.

Unlike previous studies comparing ultrasound-guided or ultrasound-assisted techniques with landmark-based approaches, the present study focused on whether modification of the puncture pathway could further improve procedural outcomes after ultrasound localization. The reduced number of needle passes observed in the modified approach group suggests that the selection of puncture site and needle trajectory may influence technical performance during spinal anesthesia.

Previous studies have demonstrated that ultrasound-assisted techniques can improve the accuracy of neuraxial procedures by facilitating identification of spinal structures and optimizing needle placement (6, 10, 16, 17). Chin et al. (6) reported that preprocedural ultrasound improved first-pass success and reduced needle manipulation in patients with potentially difficult spinal anatomy, including obesity, scoliosis, and previous lumbar surgery. Chen et al. (2), Zeng et al. (14), and Khan et al. (18) further demonstrated that ultrasound-assisted approaches could reduce needle attempts and improve procedural success compared with conventional landmark-based techniques.

However, the clinical benefits of preprocedural ultrasound may vary among different patient populations. Jiang et al. (19) reported that the improvement in first-pass success associated with ultrasound guidance was more apparent in patients with predicted puncture difficulty, whereas the benefit was limited in patients with easily identifiable anatomical landmarks. Young et al. (3) similarly suggested that the effectiveness of ultrasound-assisted neuraxial techniques may depend on patient characteristics and procedural conditions. Therefore, ultrasound should be regarded as an adjunctive technique that facilitates procedural planning rather than a universal replacement for landmark-based approaches. Further refinement of needle insertion strategies may provide additional opportunities to improve neuraxial procedural performance.

In contrast to previous investigations comparing ultrasound-assisted and landmark-guided techniques, both groups in the present study underwent ultrasound localization before puncture. Therefore, the differences observed between groups are unlikely to be explained solely by improved anatomical visualization. Instead, they may reflect the influence of puncture point selection and needle trajectory. These findings provide preliminary evidence that modifying the puncture pathway may offer additional advantages beyond ultrasound-assisted localization.

The traditional paramedian approach is widely used in spinal anesthesia, particularly when the midline approach is difficult because of anatomical variations or limited interspinous space (9–11, 14, 16, 20). However, successful paramedian puncture requires accurate estimation of needle direction, and inappropriate trajectory selection may lead to repeated needle adjustments or contact with osseous structures.

In the present study, the modified paramedian approach used a puncture point located 0.5 cm lateral to the ultrasound-defined spinal midline on the non-operative side. The target point was aligned with the upper edge of the inferior lamina, followed by needle advancement according to the modified trajectory (Figure 2A). In contrast, the traditional paramedian approach used a more lateral puncture point and an oblique needle trajectory (Figure 2B). This modification may influence the spatial relationship between the needle entry point and the target interlaminar space.

**Figure 4:**
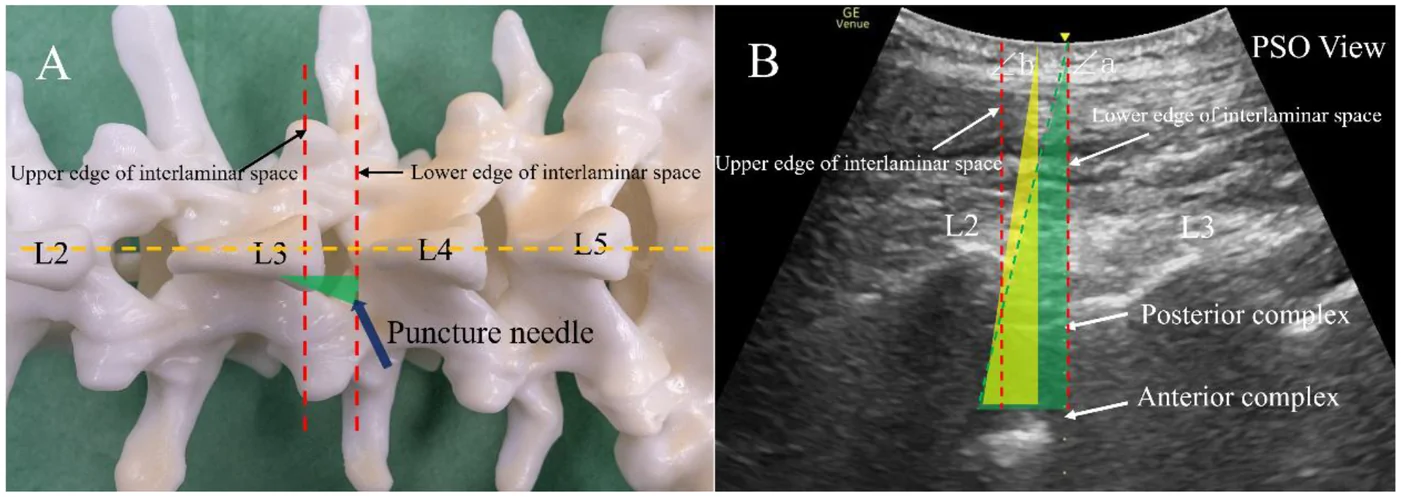
Schematic representation of the hypothesized needle advancement pathways between the modified and traditional paramedian approaches. The modified approach alters the spatial relationship between the skin entry point and the target interlaminar space compared with the traditional paramedian approach. The schematic illustrates the theoretical difference in needle advancement pathways between the two techniques.

As illustrated in Figure 4, the modified approach may theoretically provide a more favorable pathway for needle advancement by reducing lateral displacement between the entry point and target structure. This may facilitate correction of minor deviations during needle advancement and reduce unnecessary redirection. In the present study, the modified approach was associated with fewer needle passes required for successful CSF acquisition. However, because needle trajectory, insertion angle, and the spatial relationship between the needle and spinal structures were not directly measured, the proposed mechanism should be considered hypothesis-generating.

Future studies incorporating three-dimensional imaging, dynamic ultrasound assessment, or quantitative analysis of needle trajectory are required to clarify how modifications of the puncture pathway influence procedural performance.

Previous studies have emphasized the influence of anatomical characteristics on the difficulty of neuraxial access. Boon et al. (20) reported that the width of the left and right interlaminar spaces may differ, suggesting that anatomical asymmetry could affect needle advancement. In the present study, puncture sites were selected on the non-operative side, and the distribution of left and right puncture sides was comparable between groups (*P* = 0.575), reducing the potential influence of lateral anatomical differences on procedural comparison.

Although ultrasound localization was performed in all patients, first-pass failure still occurred in both groups, indicating that anatomical visualization alone does not completely eliminate technical challenges during needle advancement. Successful first-pass puncture is influenced by multiple factors, including interpretation of ultrasound images, needle control, anatomical variation, tissue resistance, and operator experience. The lower first-pass failure rate observed with the modified approach suggests that optimization of the puncture pathway may facilitate needle advancement after ultrasound localization. Nevertheless, because all patients achieved adequate anesthesia without rescue procedures, the clinical significance of improved first-pass success should be interpreted together with other procedural outcomes.

The clinical relevance of the observed differences between groups requires cautious interpretation. Although the modified approach reduced the number of needle passes and shortened procedure duration, the absolute differences were relatively small. Reduced needle manipulation may improve procedural comfort; however, whether these technical advantages translate into less puncture-related discomfort, fewer complications, improved block characteristics, or enhanced postoperative recovery remains uncertain.

Therefore, the ultrasound-assisted modified paramedian approach should be considered a refinement of the traditional paramedian technique rather than a replacement strategy. Its potential value may lie in providing a more standardized puncture pathway and improving procedural consistency, particularly in settings where ultrasound-assisted localization is routinely used.

Previous studies have reported that ultrasound image quality varies according to scanning plane, with the paramedian sagittal oblique (PSO) view providing clearer visualization of posterior spinal structures than the transverse median (TM) view (21, 22). In the supplementary analysis of the present study, the distribution of ultrasound image quality grades differed significantly between the PSO and TM views (Bowker's test of symmetry, *P* < 0.001). The PSO view demonstrated a higher proportion of clear images compared with the TM view (Supplementary Table S1).

The improved visualization obtained from the PSO view may facilitate recognition of posterior spinal structures and identification of the target interlaminar space. This may be particularly relevant for ultrasound-assisted paramedian approaches, in which accurate estimation of the puncture point and needle trajectory is required. However, the present study did not directly evaluate whether differences in ultrasound image quality were associated with procedural outcomes. Therefore, whether improved visualization independently contributes to fewer needle passes or higher first-pass success requires further investigation.

The effectiveness of a puncture strategy likely depends on the interaction between ultrasound interpretation and needle advancement technique. The potential benefit of the modified paramedian approach may therefore result from the combination of ultrasound-assisted anatomical localization and a predefined puncture pathway rather than improved visualization alone. Further studies are needed to clarify the interaction between ultrasound image characteristics, anatomical difficulty, and puncture strategies in determining neuraxial procedural performance.

Operator experience and understanding of spinal anatomy remain important determinants of neuraxial anesthesia success. For novice anesthesiologists, proficiency in spinal anesthesia requires structured training, repeated practice, and accurate understanding of the relationship between surface landmarks, ultrasound images, and needle trajectories.

Ultrasound-assisted techniques provide additional anatomical information and may serve as a useful educational tool by improving recognition of spinal structures and supporting procedural planning (23). For novice anesthesiologists, structured training integrating ultrasound interpretation with standardized needle placement strategies may facilitate the development of neuraxial anesthesia skills. Chen et al. reported that a modified paramedian technique improved puncture success among residents compared with the conventional paramedian technique, with fewer attempts required during spinal and epidural procedures (24).

For recently graduated anesthesiologists, the modified approach may provide a practical framework integrating ultrasound localization, trajectory planning, and standardized needle advancement. Nevertheless, prospective training studies are needed to determine whether this technique accelerates skill acquisition or improves long-term procedural competence.

## Limitations

Several limitations should be considered when interpreting the findings of this study.

First, this was a single-center randomized controlled trial with a relatively limited sample size, which may restrict the generalizability of the results. Although patients with American Society of Anesthesiologists (ASA) physical status I–III were eligible according to the predefined inclusion criteria, all enrolled patients were ultimately classified as ASA II because of the clinical characteristics of the recruited surgical population. In addition, patients with BMI ≥30 kg/m^2^ were excluded according to the predefined eligibility criteria. Therefore, whether the findings can be extended to patients with higher ASA physical status, obesity, or more challenging spinal anatomy remains uncertain.

Second, blinding of the anesthesiologist performing the procedure was not feasible because of the nature of the intervention. Although all procedures were performed by the same experienced anesthesiologist to minimize operator-related variability, the possibility of operator-related bias cannot be completely excluded. Furthermore, the findings may not be directly applicable to anesthesiologists with different levels of experience or to institutions with different procedural workflows.

Third, this study primarily focused on procedural performance. Detailed characteristics of spinal anesthesia quality, including block onset time, sensory block symmetry, and intraoperative analgesic effectiveness, were not systematically evaluated. Although postoperative follow-up was performed within 48 h after surgery to assess procedure-related adverse events, longer-term outcomes, including delayed complications, postoperative recovery, and persistent patient-reported outcomes, were not investigated.

Finally, patient satisfaction was assessed using a simple ordinal scale rather than a validated patient-reported outcome instrument. Although this approach was feasible for immediate postoperative evaluation, future studies using validated satisfaction questionnaires are needed to provide a more comprehensive assessment of patient experience.

## Conclusion

In this randomized controlled trial, the ultrasound-assisted modified paramedian approach was associated with fewer needle passes and a higher first-pass success rate compared with the ultrasound-assisted traditional paramedian approach for spinal anesthesia in patients undergoing lower limb surgery. The modified approach may represent a practical refinement of the puncture pathway that complements ultrasound-assisted localization while maintaining comparable short-term safety outcomes.

The observed advantages primarily reflected improvements in procedural performance, and their broader clinical significance remains uncertain. Further multicenter studies involving larger and more diverse patient populations, particularly those with difficult spinal anatomy or varying levels of operator experience, are required to determine whether this approach can provide additional clinical benefits.

## Data Availability

The raw data supporting the conclusions of this article will be made available by the authors, without undue reservation.
