# Supplementary material for: Ultrasound-assisted modified paramedian approach vs. traditional paramedian approach for spinal anesthesia in lower limb surgery: a single-center randomized controlled trial
Source: Front Med (Lausanne). 2026 Sep 3;13:1903864. doi: 10.3389/fmed.2026.1903864 (PMC13581877; doi:10.3389/fmed.2026.1903864)
Supplement: Supplementary file 1 [file Table1.docx]

**Supplementary Table S1. Comparison of ultrasound image quality between parasagittal oblique (PSO) and transverse median (TM) views**

| **Ultrasound image quality grade** | **PSO view (n = 116)** | **TM view (n = 116)** | **P value** |
| --- | --- | --- | --- |
| Clear, n (%) | 89 (76.7) | 27 (23.3) |  |
| Moderate, n (%) | 27 (23.3) | 79 (68.1) |  |
| Poor, n (%) | 0 (0.0) | 10 (8.6) |  |
| **Overall comparison** |  |  | **<0.001** |

Data are presented as number (%). Ultrasound image quality was graded as follows: grade 1 (clear), both anterior and posterior complexes visualized; grade 2 (moderate), either complex visualized; grade 3 (poor), neither complex visualized. Comparisons were performed using Bowker’s test of symmetry for paired ordinal categorical data.
